# Antiretroviral medications for pediatric use in the context of the Unified Health System: are there therapeutic gaps in Brazil?

**DOI:** 10.1590/1984-0462/2025/43/2024141

**Published:** 2025-08-04

**Authors:** Suelen Martins da Costa, Patricia Melo Aguiar

**Affiliations:** a Universidade de São Paulo, Faculdade de Ciências Farmacêuticas – São Paulo (SP), Brazil.

**Keywords:** Antiretrovirals, HIV, Pediatrics, Drugs essential, Antirretrovirais, HIV, Pediatria, Medicamentos essenciais

## Abstract

**Objective:**

To evaluate the incorporation and adequacy of antiretrovirals for the treatment of HIV in children within the context of the Unified Health System in Brazil.

**Methods:**

Data were collected from the 2022 edition of the Brazilian National List of Essential Medicines and compared with the World Health Organization’s 2023 Essential Medicines List for Children. Furthermore, records from the Brazilian Health Regulatory Agency were reviewed for new medications and pediatric formulations with the potential to fill identified gaps.

**Results:**

Twenty-one antiretrovirals are listed in the latest edition of the National List of Essential Medicines, of which 16 are suitable for pediatric use. These formulations are available predominantly in tablets (44.4%), oral solutions (22.2%), and capsules (14.8%). Compared to the World Health Organization list, the Brazilian list offers more pediatric options, including, for example, efavirenz (removed from the international list) and tipranavir. The only medication included on the international list that is registered in Brazil but not listed on the national list is the 25 mg chewable tablet of raltegravir. Recently, the Brazilian Health Regulatory Agency registered six new antiretroviral medications, with only one being child-friendly (dolutegravir) and it was incorporated into the Unified Health System.

**Conclusions:**

The comparison between the national and international essential medicines lists highlights the greater variety of pediatric antiretrovirals in Brazil but underscores the need for more child-friendly formulations. The implementation of protocols for the manipulation and fractionation of solid pharmaceutical forms is urgent in order to improve treatment adherence and therapeutic efficacy.

## RESUMO

**Objetivo:**

Avaliar a incorporação e adequação dos antirretrovirais para o tratamento do HIV em crianças no contexto do Sistema Único de Saúde no Brasil.

**Métodos:**

Os dados foram coletados da Relação Nacional de Medicamentos Essenciais 2022 e comparados com a Lista de Medicamentos Essenciais para Crianças de 2023 da Organização Mundial da Saúde. Além disso, os registros da Agência Nacional de Vigilância Sanitária foram revisados para novos medicamentos e formulações pediátricas com potencial para preencher as lacunas identificadas.

**Resultados:**

Vinte e um antirretrovirais estão listados na edição mais recente da Relação Nacional de Medicamentos Essenciais, sendo 16 adequados para uso pediátrico. As principais apresentações disponíveis são comprimidos (44,4%), soluções orais (22,2%) e cápsulas (14,8%). Comparada à lista da Organização Mundial de Saúde, a lista brasileira oferece mais opções pediátricas, incluindo, por exemplo, o efavirenz (removido da lista internacional) e o tipranavir. O único medicamento incluído na lista internacional que está registrado no Brasil, mas não consta na relação nacional, é o comprimido mastigável de raltegravir de 25 mg. Recentemente, a Agência Nacional de Vigilância Sanitária registrou seis novos antirretrovirais, mas apenas um é adequado para crianças (dolutegravir) e foi incorporado no Sistema Único de Saúde.

**Conclusões:**

A comparação entre as listas nacionais e internacionais de medicamentos essenciais destaca a maior variedade de antirretrovirais pediátricos no Brasil, mas sublinha a necessidade de mais formulações adequadas para crianças. A implementação de protocolos para a manipulação e fracionamento de formas farmacêuticas sólidas é urgente para melhorar a adesão ao tratamento e a eficácia terapêutica.

## INTRODUCTION

 The use of medications in pediatrics represents a global challenge. The scarcity of new technologies and the lack of investment in clinical studies to meet the demands of this population leads to the prescription of formulations without therapeutic indication for the age group.^1^ It is estimated that two-thirds of pediatric patients worldwide are treated with medications that are not indicated for their age,^2^ which is especially concerning for neglected diseases, since the limited therapeutic options impact both children and adults.^3^

 The Brazilian Health Regulatory Agency (ANVISA), responsible for the approval and registration of new medications, does not have specific norms or guidelines for the use of pediatric medicines, nor has it established protocols for off-label prescription.^3^ The demand for regulations has been recognized for decades and is part of the National Medication Policy established in 1998, which emphasizes the need to "ensure the access of drugs in pharmaceutical forms and dosages appropriate for specific population groups, such as children and the elderly".^4^ However, data from clinical trial registries reveal a limited participation of children globally in drug-related studies, with this age group representing less than 8% of the total.^1^ Additionally to the reduced number of clinical trials conducted with children, some of the studies performed by multinational companies in Brazil may also investigate pharmaceutical forms unsuitable for pediatrics.^5^

 Incorporating essential medicines into standardized lists and ensuring access to appropriate treatments are important factors in pediatric care. In 2020, around 5 million children under the age of 5 died from preventable causes that have available treatments, and 2.4 million of these deaths were among newborns up to 28 days old,^6^ underscoring the importance of access to essential medications. In this context, the List of Essential Medicines for Children (EMLc) was created by the World Health Organization (WHO) in 2007, as a tool to highlight the essential components necessary for a healthcare system to meet the needs of children, which is critical for the United Nations. It does not replace the national lists assembled by each country but it is an informative guide that contemplates the most effective, safest, and cost-effective medications for priority conditions.^7^

 Most countries do not have a specific list of medications for children but they aim to keep their lists "child-friendly" by adding pediatric formulations, as well as products indicated for both adults and children.^8^ Brazil aligns with this trend by consolidating its essential, specialized, and strategic components of Pharmaceutical Assistance in the National List of Essential Medicines (RENAME),^9^ which serves as an important guiding instrument for the use of medications and supplies within the Unified Health System (Brazil’s public health care system). 

 Human immunodeficiency virus (HIV) infection, in particular, is considered a high-priority neglected condition when it comes to children, given the shortage of studies and treatment alternatives.^10^ In Brazil, around 54,804 children were exposed to HIV between 2015 and 2022, with most cases in newborns under 7 days old.^11^ Although HIV vertical transmission rates have significantly decreased over the years with appropriate maternal care,^12^ statistics remain concerning. The United Nations Children’s Fund reported that in 2020, at least 300,000 children worldwide were newly infected with HIV.^13^ In Latin America, only about 40% of children aged 0 to 14 years living with HIV have access to antiretroviral therapy.^14^ Also, only one-third of HIV-seropositive children and adolescents have adherence levels above 95%, a percentage considered ideal for effective HIV treatment.^15^

 The administration of antiretroviral therapy to children aims to reduce morbidity and mortality and improve quality of life through viral suppression, which helps delay the onset of immunodeficiency. Protocols for pediatric HIV management recommend that the initial antiretroviral regimen must be structured with three antiretroviral drugs: two nucleoside reverse transcriptase inhibitors and a third from a different therapeutic class (according to the patient’s age), such as non-nucleoside reverse transcriptase inhibitors, protease inhibitors, or integrase inhibitors.^16^ In addition, body mass, sexual maturation rate, and genotyping results are other factors to consider when choosing the therapeutic strategy.^17^ However, inadequate and complex pharmaceutical presentations can affect both adherence and efficacy of therapy for child treatment, and despite efforts to reduce the obstacles in the treatment, caregivers still have difficulties handling dosages, and not all currently available medications are palatable or easily swallowed.^15,18^

 This study aimed to assess the incorporation of antiretrovirals into the RENAME and their adequacy for treating HIV in children when compared to the latest edition of the EMLc recommended by the WHO, as well as to evaluate possible therapeutic gaps and perspectives for solutions in Brazil. 

## METHOD

 An analysis of the 2022 RENAME^9^ was conducted to identify the antiretroviral medicines incorporated into Brazil’s Unified Health System. These medications were compared with the latest EMLc edition, recommended by WHO^7^ , and with items registered by ANVISA, but unavailable in the Brazilian public health system. Both active ingredients and formulations with indications in the package insert for individuals aged 0 to 12 years, or with no documented age restriction, were considered without distinction between antiretrovirals intended for the treatment of HIV, Pre-Exposure Prophylaxis, or Post-Exposure Prophylaxis. Medications for exclusive use in adults, absent from protocols for managing HIV infection in children, or lacking sufficient literature about their pediatric use were not considered for the analysis. 

 Information on antiretrovirals not listed in the Brazilian public health system was located through a manual search on the ANVISA website, utilizing the fields of therapeutic class ("antiretrovirals", "antivirals", "antivirals for systemic use") and/or Anatomical Therapeutic Chemical (ATC) classification ("combinations of antivirals for the treatment of HIV infections", "antivirals for systemic use", "antivirals", "antivirals"). Multiple registrations of the same medicine were also checked using the name of the drug’s active or commercial ingredient. 

 The collected data were classified according to the name of the active ingredient, therapeutic class (according to the 4th level of ATC classification), pharmaceutical form, and route of administration. Information on pediatric use in the treatment of HIV and age-related restrictions on use were also identified. Data were presented descriptively. 

## RESULTS

 A total of 21 medicines classified as antiretrovirals were identified in the latest published edition of RENAME, 16 products available for pediatrics (76.2%; Table 1) and five for exclusive use in the treatment of adults (23.8%; Table 2). Among the medicines with indication for pediatric use, 27 pharmaceutical presentations were found distributed among tablets (44.4%), oral solutions (22.2%), capsules (14.8%), oral suspensions (7.4%), chewable tablet (3.7%), injectables (3.7%), and powders for injectable solutions (3.7%). Regarding the recommendation for use by age, only nevirapine, ritonavir, and zidovudine are exempt from restrictions. The others are restricted mainly to ages younger than three or six months. 

**Table 1:** Antiretrovirals and their pharmaceutical presentations available for pediatric use listed in the Brazilian National List of Essential Medicines (RENAME), classified by therapeutic class.

Therapeutic class 4th level ATC	Antiretroviral	Pharmaceutical presentation	Age restriction
Protease Inhibitor	Darunavir	Tablet 75/150/600 mg	≥6 years old; with experience in antiretroviral treatment
	Fosamprenavir	Oral Suspension 50 mg/mL	≥4 weeks
	Ritonavir	Tablet 100 mg	≥4 weeks
	Atazanavir Sulfate	Capsule 300 mg	≥6 weeks
Non-nucleoside Reverse Transcriptase Inhibitors (NNRTIs)	Efavirenz	Tablet 600 mg	≥3 years of age; weighing 13 kg or more
		Capsule 200 mg	
		Oral Solution 30 mg/mL	
	Nevirapine	Tablet 200 mg	No restriction
		Oral Solution 30 mg/mL	
Integrase Inhibitors	Dolutegravir sodium	Tablet 50 mg	≥12 years old; weighing more than 40 kg
	Raltegravir Potassium	Chewable Tablet 100 mg	≥2 years
		Tablet 400 mg	≥6 years
Fusion Inhibitor	Enfuvirtide	Powder for Injectable Solution 90 mg/mL	≥6 years
Nucleotide analogue reverse transcriptase inhibitor (NRTIs)	Lamivudine	Oral Solution 10 mg/mL	≥3 months
		Tablet 150 mg	≥3 months; weighing 14 kg or more
	Abacavir Sulfate	Oral Solution 20 mg/mL	≥3 months
		Tablet 300 mg	≥3 months; weighing 14 kg or more
	Zidovudine	Capsule 100 mg	No restriction
		Solution for Injection 10 mg/mL	
		Oral Solution 10 mg/mL	
Non-peptide protease inhibitor	Tipranavir	Soft Capsule 250 mg	≥2 years
		Oral Solution 100 mg/mL	
Fixed-dose antiretroviral combination	Lopinavir + Ritonavir	Tablet 200 mg + 50 mg		≥6 months; no swallowing problems
		Tablet 100 mg + 25 mg		
		Oral Solution 80 mg/mL + 20 mg/mL	≥6 months
	Tenofovir disoproxil fumarate + lamivudine + efavirenz	Tablet 300 mg + 300 mg + 600 mg	3 to 12 years
	Zidovudine + lamivudine	Tablet 300 mg + 150 mg	≥12 years

ATC: Anatomical Therapeutic Chemical.

**Table 2:** Antiretrovirals and their pharmaceutical presentations not available for pediatric use listed in the Brazilian National List of Essential Medicines (RENAME), classified by therapeutic class.

Therapeutic Class4th level ATC	Antiretroviral	Pharmaceutical presentation
Non-Nucleoside Reverse Transcriptase Inhibitors (NNRTIs)	Etravirine	Tablet 100/200 mg
Nucleotide analogue reverse transcriptase inhibitor (NRTIs)	Tenofovir disoproxil fumarate	Tablet 300 mg
CCR5 co-receptor antagonist	Maraviroc*	Tablet 150 mg
Fixed-dose antiretroviral combinations	Tenofovir disoproxil fumarate + emtricitabine	Tablet 300 mg + 200 mg
	Tenofovir disoproxil fumarate + lamivudine	Tablet 300 mg + 300 mg

* Registered by the Brazilian Health Regulatory Agency only for adult use, but included in the Clinical Practice Guideline for Pediatric HIV management^16^ and approved for use in children aged 2 years and older, in other countries.

ATC: Anatomical Therapeutic Chemical.

 Compared to the WHO EMLc, RENAME has a larger arsenal of pediatric antiretrovirals. There are 16 active ingredients on the Brazilian list, while WHO highlights ten as essential and all of them are distributed by the Brazilian public health system. Among the 16 drugs listed in RENAME, three were removed from previous editions of the WHO EMLc (abacavir sulfate, efavirenz, and atazanavir). The drugs tipranavir, fosamprenavir, enfuvirtide, and maraviroc found in RENAME are not present in the latest edition of the international EMLc and are not mentioned in any of the previous editions. In all, there are 15 dosage forms compiled by the WHO, seven tablets (46.6%), three oral solutions (20.0%), three dispersible solid formulations (20.0%), one chewable tablet (6.7%), and one granule for suspension (6.7%); 46.7% of the pharmaceutical forms presented in the WHO EMLc are not available through the Brazilian HIV program, and only the 25 mg chewable tablet of raltegravir was registered by ANVISA (Table 3). 

**Table 3:** Antiretrovirals and their pharmaceutical presentations listed as Essential Medicines for Children according to the World Health Organization and their inclusion in the Brazilian National List of Essential Medicines (RENAME).

Therapeutic Class4th level ATC	Antiretroviral	Pharmaceutical presentation	Listed in RENAME?
Nucleoside/nucleotide reverse transcriptase inhibitor (NNRTIs)	Lamivudine	Oral Solution 50 mg/5 mL	Yes
	Zidovudine	Oral Solution 50 mg/5 mL	Yes
Non-nucleoside reverse transcriptase inhibitor (NRTIs)	Nevirapine	Oral Solution 50 mg/5 mL	Yes
		Dispersible Tablet 50 mg*	No
Protease inhibitor	Darunavir	Tablet 75 mg	Yes
	Lopinavir + ritonavir	Solid Oral 40 mg + 10 mg*	No
		Thermostable Tablet 100 mg + 25 mg	Yes
	Ritonavir	Thermostable Tablet 25 mg*	Yes
		Thermostable Tablet 100 mg	Yes
Integrase inhibitor	Dolutegravir	Dispersible Tablet 10 mg*	No
		Tablet 50 mg	Yes
	Raltegravir	Granules for Suspension 100 mg*	No
		Chewable Tablet 25 mg†	No
Fixed-dose combinations of antiretrovirals	Abacavir + Lamivudine	Dispersible Tablet 120 mg (as sulfate) + 60 mg*	No
	Lamivudine + Zidovudine	Tablet 30 mg + 60 mg*	No

* Not registered by the Brazilian Health Regulatory Agency

† Registered by the Brazilian Health Regulatory Agency

ATC: Anatomical Therapeutic Chemical.

 Among the registrations of antiretroviral drugs indicated for the treatment of HIV within ANVISA, which are not part of RENAME or the strategy to combat HIV (Table 4), six drugs have recent registrations (occurred in the last five years) and are new products, except for Tivicay PD, which is a new presentation of dolutegravir sodium more suitable for children (tablet in suspension, 5 mg). Apretude (cabotegravir) was approved for registration in June 2023 and is the only long-acting injectable on the list, as well as the only one indicated for Pre-Exposure Prophylaxis in adults and adolescents. The remaining drugs, Symtuza, Biktarvy, Dovato, and Juluca, are innovative combinations of antiretrovirals marketed as complete regimens for the treatment of HIV, but only Biktarvy has an indication for children. 

**Table 4:** Antiretroviral medications indicated for the treatment of human immunodeficiency virus registered by the Brazilian Health Regulatory Agency not listed in the Brazilian National List of Essential Medicines (RENAME).

Product	Active ingredient	Age indication	Pharmaceutical presentation
Symtuza	Darunavir + cobicistate + emtricitabine + tenofovir alafenamide	Children over 12 years old	Coated Tablet
Biktarvy	Bictegravir + emtricitabine + tenofovir alafenamide	Children over 6 years old weighing at least 25 kg	Coated Tablet
Tivicay PD*	Dolutegravir sodium	Children over 4 weeks > 4 kg	Tablet for Suspension
Apretude	Cabotegravir	Individuals weighing at least 35 kg	Tablet & Injectable
Juluca	Dolutegravir sodium + rilpivirine hydrochloride	Virologically suppressed adult subjects for at least 6 months	Coated Tablet
Dovato	Dolutegravir + Lamivudine	Individuals weighing at least 40 kg and over 12 years old	Coated Tablet

* Incorporated in the latest Clinical Practice Guideline for Pediatric HIV management16 published by the Brazilian Health Ministry, but not yet officially listed in the 2022 RENAME (possibly in the next updated version).

## DISCUSSION

 Brazil was the first country worldwide to ensure universal access to antiretroviral therapy, and one of the first to implement the policy of free distribution of disposable needles and syringes, with a strong approach to combating stigma and a focus on information and prevention.^19^ It was also a pioneer both in the production of generic drugs and in the negotiation with the pharmaceutical industry to reduce the prices of antiretrovirals. In 2007, Brazil adopted compulsory licensing of these medicines. This combination of factors contributed to turning the Unified Health System into a recognized model in the global fight against the HIV epidemic, inspiring initiatives by the WHO alongside the Joint United Nations Programme on HIV/AIDS (UNAIDS) in 2003 to ensure access to antiretroviral therapy in poor countries.^19^

 The legacy of the Brazilian government’s actions remains evident in current clinical practice guideline and list of medicines. RENAME includes all antiretroviral drugs listed in the international EMLc, regardless of the availability of the same forms of presentation suggested by the WHO, evidencing the investment in resources for accessing these treatments. In comparison, countries like India might need to update their essential medicine lists to enhance access to pediatric HIV treatments.^20^ Although the WHO EMLc has fewer antiretrovirals than RENAME, it features more child-friendly options (from neonatal age), highlighting its role as a tool for suggesting essential medications for children. 

 Both lists reveal the scarcity of treatment options for newborns aged between 14 days and one month. There are only two possible combinations recommended, with the preferred third antiretroviral options being raltegravir or lopinavir with a ritonavir booster, due to the frequent exposure of children to nevirapine while still in utero and the increased risk of antiretrovirals resistance.^16^ Additionally, when including the rescue schemes for treatment failure, the possibilities for children under 6 years old are also quite limited.^16^ However, for children over 6 years, the RENAME enables the use of a sequential antiretroviral strategy upon therapeutical failure, including fusion inhibitor (enfuvirtide) and CCR5 inhibitor (maraviroc) — drugs not listed on the WHO EMLc. It’s worth noting that therapeutic failure is very common in children^21^ due to inadequate strategies, such as monotherapy and dual therapy, which often lack sufficient potency against viral replication and present a low genetic barrier to resistance.^22^ Yet, those often become the only viable options due to the lack of response to other drugs. 

 It should be noted that tablets are the main pharmaceutical presentation of antiretrovirals available to children, although many have difficulty swallowing medications. Typically, as early as age 10, they are able to start swallowing pills, and it is possible to train children as young as 5 years old to swallow.^23,24^ Despite this, some medications on both the WHO EMLc and RENAME are only available as tablets, even though they are indicated for children under 3 years. An example is the darunavir, which requires intervention prior to administration, such as dissolving the tablet — an additional step that might increase the difficulty of an already complex treatment regimen due to the combination of medicines involved,^25^ escalating the likelihood of administration errors. Overall, liquid dosage forms for oral use are more suitable for pediatric care, due to their ease of use and rapid dose adjustment.^1^ However, there is a preference in the production of solid forms due to their stability, palatability, and lower number of excipients required for their formulation.^7^ Daily oral treatments remain the most significant barrier to long-term suppression of virus replication and prevention, as even short periods of nonadherence may endanger patients’ health and narrow their choices for future treatment, with the emergence of drug-resistant virus variants.^26^

 The most recent innovations in antiretrovirals are complete single-dose regimens, which allow people living with HIV to take a single drug once a day. One such drug is the 4-in-1 granule capsule, developed by the Drugs for Neglected Diseases initiative (DNDi) in partnership with Cipla, a combination of heat-resistant, strawberry-flavored lamivudine and ritonavir with lamivudine and abacavir;^27^ another is the 2-in-1 formulation on pallets of two associated nucleoside reverse transcriptase inhibitors for patients who are unable to swallow tablets.^28^ In Brazil, a group from the Oswaldo Cruz Foundation (Fiocruz) has been working since 2016 on the development of an orodispersible formulation of efavirenz based on nanotechnology, to reduce the rejection of the taste of the oral solution.^29^ The formulations are promising, but still, there are no estimated dates for registry or release in Brazil. 

 Broadly neutralizing antibodies, microarray patches, and subcutaneous formulations for prophylaxis and treatment in neonates are some of the products of potential interest for pediatric treatment in the future.^30^ These long-acting or extended-release formulations have multiple advantages over daily oral therapy, including potential for reducing laboratory monitoring and improving adherence, before avoiding pill fatigue and stigma.^31^

 Among the components registered in Brazil over the past five years but not included in the RENAME list, one lacks the pediatric indication (Juluca) and another is not recommended for incorporation by the National Committee for the Incorporation of Technologies in the Unified Health System (Conitec)^32^ due to insufficient clinical evidence. Cabotegravir had its registration approved in the country in June 2023 but has yet to undergo technological evaluation. Tivicay PD (dolutegravir sodium 5 mg) was the only product on the list with a favorable opinion from Conitec for its use as a complementary or substitute treatment in children with HIV aged from 2 months to 6 years, justified by the need to address the health needs of a population that is difficult to manage therapeutically.^33^ The recent updates for the Brazilian pediatric protocol of people living with HIV now include Tivicay as a possibility for the treatment of children over 3 kg and older than 4 weeks, with the dosage specified according to the number of tablets that might be dispersed for daily administration.^16^

 The development of dispersible solid formulas with flexible dosages, such as tablets that can be dissolved in beverages before administration, has been recommended by the WHO as a viable option for antiretrovirals,^34^ with dolutegravir 5 mg recently incorporated into the Brazilian public health system. Once the drug is dispersed in liquid, it can be easily swallowed by the pediatric population, in addition to being an indication of interesting value for underdeveloped countries. The main disadvantage is the limited flexibility of fractionation, which requires more than one dosage of the same drug to be available.^34^

 The availability of suitable antiretroviral medications and formulations for preventing and treating HIV in children and adolescents has traditionally lagged 7–12 years behind that of adults,^30^ making the derivation, crushing and spraying of pills, or removal of capsule contents — a reality that cannot be avoided. In this context, the adaptation of the formulas is carried out by the patient himself or his caregiver and, due to its chronic nature, the practice of adaptation is repeated daily, considerably increasing the risk of medication errors over the years. Therefore, it is crucial to establish specific guidelines on the handling of common medicines in pediatrics, the systematic inclusion of pediatric demands in the health technology assessment process,^1^ as well as the qualified guidance of caregivers. 

 It is important to acknowledge that this study mainly focused on the standardization of antiretrovirals in RENAME and did not directly evaluate their access at the point of care within the Brazilian public health system. This limited focus restricts the analysis to identifying which essential components are still lacking for adequate pediatric HIV management, without providing insights into their availability or how efficiently they cover the populational needs of care. Thus, future research should investigate not only the incorporation of these medicines but also their integration into clinical practice and their access in healthcare settings — considering availability, acceptability, and geographic accessibility aspects.^35^ This broader perspective will yield a more comprehensive understanding of pediatric HIV management in Brazil and the ways to improve antiretroviral therapy access. 

 In conclusion, the arsenal of pediatric antiretrovirals incorporated into RENAME is wider than that of the WHO EMLc. However, the pharmaceutical presentations found on RENAME diverge from those recommended by the WHO. Most of them are not even registered by ANVISA, evidencing a therapeutic gap in RENAME, especially when it comes to orodispersible options. Consequently, inadequate presentations for children require adaptations often related to medication errors, a situation intensified in chronic diseases. Inadequate management of antiretroviral therapy can also lead to resistance, decreasing the availability of already restricted HIV therapeutic combinations. Strategies for the proper handling of drugs are especially relevant in developing countries, where health infrastructure may be insufficient to fully meet the specific needs of the pediatric population. 

## Data Availability

The database that originated the article is available with the corresponding author.
